# Extraction of Deciduous Teeth

**Published:** 1883-08

**Authors:** N. W. Kingsley

**Affiliations:** New York


### Independent Practitioner.

VOL. IV. AUGUST, 1883. No. 8.

### Original Communications.

### EXTRACTION OF DECIDUOUS TEETH.

BY N. W. KINGSLEY, D. D. S., NEW YORK.

WHITNEY MEMORIAL PRIZE ESSAY. PRESENTED BEFORE THE DENTAL SOCIETY OF THE STATE OF NEW YORK, MAY, 1883.

Quite recently there have appeared in the reports of papers and discussions before dental societies, opinions expressed upon the extraction of the deciduous teeth which are so much at variance with the author's experience and observations, that he ventures to put his views in the form of an essay.

The views which he deems erroneous have shown a misconception of the order in which the temporary teeth are shed and their places supplied by permanent ones, and also in the idea which has been entertained that the premature removal of the deciduous teeth caused a shrinkage of the jaw, and created an irregularity in the permanent dental arch.

It would hardly seem possible, in view of the researches of the last quarter of a century, that any educated dentist could hold any other than one opinion, or that it should be necessary at this time to correct any erroneous impressions. Two or three illustrations will serve to show the errors alluded to.

A gentleman of well known eminence in the profession was describing before one of the societies his method of caring for children's teeth, when he said among other things, "that as soon as there was any evidence of the eruption of a permanent tooth he removed the deciduous tooth to give it room. Beginning with the central incisors, which were the first to make their appearance, if the removal of the centrals did not promise ample room for their successors then he also took out the lateral incisors. If when the lateral incisors emerged there appeared to be a lack of room, he removed the temporary canine—following this, if the permanent canine when it appeared showed a want of space, he removed the first temporary molar, and in like manner, and in their turn according to his plan, the second temporary molar was removed to make room for the first bicuspid, and finally, if there was not room in the dental arch for the second bicuspid he extracted it, and by this course of procedure he was always able to secure a perfectly regular dental arch in the second set, or permanent teeth."

This is the teaching of one who holds no mean place in the estimation of his fellow practitioners.

Another gentleman, occupying also a prominent place in dental societies, says that in his treatment of children's teeth, when the time arrives for the shedding of the temporary tooth he extracts it to give the permanent tooth a chance, regardless of any evidence that the permanent tooth is ready to erupt. He extracts when the period arrives for the permanent tooth to erupt, because he holds that its eruption is retarded by the continued presence of the temporary one. And again, another gentleman whose exalted attainments have been conceded for a generation, maintains that the premature extraction of temporary teeth involves a contraction of the jaws. It is not an error in the use of terms that he makes (using the word jaws when he means alveolar processes), but he refers to a contraction of the jaw bones themselves.

These examples are sufficient to illustrate the object of this paper, for in the opinion of the writer each and all are erroneous.

Beginning with the last, it is a settled fact that the development of jaw bones and alveolar processes are entirely independent operations of nature.

There is a period in the history of the jaw when it is as much a jaw bone as at any subsequent time, and before a tooth has made any appearance. If through any freak of nature no tooth ever develops, the jaw will in no respect be aborted in its growth. It will continue to thicken and elongate in the case of the lower one, and widen and enlarge in the case of the upper, until it has reached the full measure of its inherited type, and neither the absence of teeth congenitally nor their removal after development will interfere with this function.

But in the growth of the alveoli as a process of the jaw we find an entirely different condition.

Alveolas are the result of the development of the teeth and coincident with their growth. The alveolar processes to a certain extent are constantly changing. It is quite doubtful if a single bony particle of the alveoli of adult life formed a part of the alveoli of childhood.

In a purely physiological condition it is forming and absorbing — forming again and again absorbing—and again a third time forming, only to be absorbed again when the final issue arrives of the loss of the teeth.

In a pathological condition this process of formation and absorption may go on repeatedly.

The mistaken idea of the shrinkage of the jaws must certainly be based upon changes which are apparent in alveolar processes, but which *do not involve the jaw.*

That the premature extraction of the deciduous teeth involves a contraction of the jaws is a mistake, and to a limited extent it is a misconception that such extraction will involve such contraction of the alveolar arch as will induce irregularity, either in the period or order of eruption, or the arrangement of the permanent set. This is true as applied to sixteen out of twenty teeth that make up the complement of the deciduous set.

The order of shedding and of eruption shows that the first to change places are the central incisors; secondly, the lateral incisors; thirdly, *not* the canines, but, frequently the second molar for the second bicuspid; fourthly, the remaining molar for the other bicuspid, and lastly the canines.

In a normal condition the jaw bones *continue* their growth *after* the growth of process about the temporary teeth has ceased, and thus as the period approaches for the eruption of the permanent teeth we find spaces between the temporary ones, thus enlarging the alveolar arch for the accommodation of the larger members of the permanent group.

The growth or enlargement of that part of the jaw upon which the deciduous dental arch is situated seems to have obtained its complete development at the period of shedding, and the incisors and bicuspids will find room equal to their necessities.

The premature extraction of any or all even of these eight named teeth, will not interfere with the natural and expected enlargement of the jaw, but the premature extraction of the canine teeth will be likely to lead to most serious results.

After the jaw bone has ceased its enlargement there seems an almost universal tendency for the bicuspids and molars to crowd to the anterior part of the mouth, and to fill any space in the alveolar arch that may not already be occupied. This is not only true in the formative period, but is equally true in adult life, if the occlusion of the opposing jaw does not counteract it. The consequence of this inevitable tendency is, that unless the temporary canines remain in their places until their permanent successors are ready to emerge, the bicuspids and whatever molars are behind them will crowd forward and occupy the space which belongs to the canines.

We thus see that whatever may be the inducement to remove any or all of the deciduous teeth prior to their period of shedding, the canines should be retained until there is ample evidence of the early emergence of their permanent successors, unless the health and comfort of the child would be sacrificed in so doing. But it would be far better to remove one or all of the deciduous teeth and take the risks of irregularity in the permanent ones, than to submit the child to constant suffering and consequent injury to its health by their retention.

In a case of retarded dentition the writer takes issue with the practitioner who removes the deciduous teeth when the usual period arrives for shedding, regardless of any evidence that the permanent ones are ready to erupt. *His* reason is, that the retention of the temporary tooth retards the growth of the permanent one.

In this issue is involved the function of absorption, and his practice would indicate that the non-absorption of the temporary tooth was the primal cause of the retarded dentition, rather than that retarded dentition is the cause of the non-absorption. Cause and effect are in his mind evidently transposed.

Such a practice will unquestionably lead in many cases to serious results.

It is not always certain, when there is no outward indication, that a tooth lies concealed.

It is not a very uncommon thing to see some one tooth of the permanent set missing, and to learn that it never erupted. And again, a retarded dentition generally indicates teeth of better organization and less liable to decay than those which have developed at an earlier age.

So long as deciduous teeth remain in the jaw in a firm and undecayed condition, with no evidence of a misdirection of their permanent successors, it is not advisable to remove them.

